# The health status of Q-fever patients after long-term follow-up

**DOI:** 10.1186/1471-2334-11-97

**Published:** 2011-04-18

**Authors:** Gabriëlla Morroy, Jeannette B Peters, Malou van Nieuwenhof, Hans HJ Bor, Jeannine LA Hautvast, Wim van der Hoek, Clementine J Wijkmans, Jan H Vercoulen

**Affiliations:** 1 Department of Infectious Disease Control, Municipal Health Service Hart voor Brabant, 's-Hertogenbosch, the Netherlands; 2 Academic Collaborative Centre AMPHI, Department of Primary and Community Care, Radboud University Nijmegen Medical Centre, the Netherlands; 3 Department of Medical Psychology, Radboud University Nijmegen Medical Centre, the Netherlands; 4 Department of Pulmonary Diseases, Radboud University Nijmegen Medical Centre, the Netherlands; 5 Centre for Infectious Disease Control, National Institute for Public Health and the Environment, Bilthoven, the Netherlands

**Keywords:** Q-fever, Coxiella burnetii, cohort, integral health status, quality of life

## Abstract

**Background:**

In the Netherlands, from 2007 to 2009, 3,522 Q-fever cases were notified from three outbreaks. These are the largest documented outbreaks in the world. Previous studies suggest that symptoms can persist for a long period of time, resulting in a reduced quality of life (QoL). The aim of this study was to qualify and quantify the health status of Q-fever patients after long-term follow-up.

**Methods:**

870 Q-fever patients of the 2007 and 2008 outbreaks were mailed a questionnaire 12 to 26 months after the onset of illness. We assessed demographic data and measured health status with the Nijmegen Clinical Screening Instrument (NCSI). The NCSI consists of three main domains of functional impairment, symptoms and QoL that are divided into eight sub-domains. The NCSI scores of Q-fever patients older than 50 years (N = 277) were compared with patients younger than 50 years (N = 238) and with norm data from healthy individuals (N = 65) and patients with chronic obstructive pulmonary disease (N = 128).

**Results:**

The response rate was 65.7%. After applying exclusion criteria 515 Q-fever patients were included in this study. The long-term health status of two thirds of Q-fever patients (both younger and older than 50 years) was severely affected for at least one sub-domain. Patients scores were most severely affected on the sub-domains general QoL (44.9%) and fatigue (43.5%). Hospitalisation in the acute phase was significantly related to long-term behavioural impairment (OR 2.8, CI 1.5-5.1), poor health related QoL (OR 2.3,CI 1.5-4.0) and subjective symptoms (OR 1.9, CI 1.1-3.6). Lung or heart disease, depression and arthritis significantly affected the long-term health status of Q-fever patients.

**Conclusions:**

Q-fever patients presented 12 to 26 months after the onset of illness severe -clinically relevant- subjective symptoms, functional impairment and impaired QoL. All measured sub-domains of the health status were impaired. Hospitalisation and co-morbidity were predictors for worse scores. Our data emphasise that more attention is needed not only to prevent exposure to Q-fever but also for the prevention and treatment of the long-term consequences of this zoönosis.

## Background

Q-fever is a worldwide zoönotic disease caused by *Coxiella burnetii *(C. burnetii), an obligate intracellular bacterium. Until 2007 Q-fever was uncommon in the Netherlands, with 5-20 notified cases annually [1]. From 2007-2009, 3,522 cases were notified in three large outbreaks [2], with dairy goats implicated as the source [1,2]. The majority of Q-fever patients (80%) reside in the southern province of Noord-Brabant [1-3]. Between 2007 and early 2010 some hard-hit communities suffered a cumulative incidence of 2,650 notified Q-fever cases per 100,000 inhabitants (one in 38 people).

In general 60% of infected Q-fever patients are asymptomatic, while 20% develop mild symptoms [4]. The remaining 20% of Q-fever patients present with more severe symptoms ranging from high fever, severe headache, night sweating, nausea and diarrhoea, to pneumonia, hepatitis, pericarditis, myocarditis and neurological symptoms [5]. Chronic Q-fever may develop in 1.5-5% of acute cases, due to reactivation of C. burnetii [4,6,7]. A feared complication is endocarditis, which may take 10-15 years to develop. In particular pregnant women and patients with heart valve disorders, vascular prosthesis and impaired immunity have a higher risk to develop chronic infection [4,6,7]. Protracted fatigue up to 10 years after infection [8,9] is another late sequel. A *Post-Infection Fatigue Syndrome *(PIFS) [9] may also occur after other infections such as Lyme disease [10]. In 10-15% of Q-fever patients fatigue can last up to 5-10 years [11] and is referred to as *Post Q-fever fatigue Syndrome *(PQFS). Other authors [8,9] state higher percentages of fatigue. PQFS presents with symptoms resembling those of *Chronic Fatigue Syndrome *(CFS).

During the Dutch Q-fever outbreaks patients and general practitioners (GPs) repeatedly reported persisting symptoms to the public health authorities and in particular about fatigue. These signals could not be substantiated, as we lacked specific information on the health status at individual and at Q-fever patient population level. Furthermore, we were uncertain whether data from other small national [12] and international studies, would also apply to our large Dutch Q-fever cohorts. In order to assess the long-term health status of Dutch Q-fever patients we started this study.

Long-term health status impairment may have a large impact on patients, their families and the societies that they are part of. In this study, the primary aim was to provide a detailed assessment of the health status of Q-fever patients 12 to 26 months after the onset of illness. This information will assist clinicians and patients to better understand the natural course, consequences of the disease and predictors for an affected health status.

## Methods

### Q-Quest I study

This cohort study is part of the collaborative Q-Quest I study, which aims to measure the impact of the Q-fever outbreaks in terms of population health and societal implications. The study started in May 2008 and includes studies on diagnostics, treatment, clinical symptoms, costs and the long-term health status.

### Study design and population

Eligible for inclusion in this study were Q-fever patients notified in 2007 and 2008 to the Municipal Health Service "Hart voor Brabant" and "Brabant Zuid-Oost" with a first day of illness in 2007 or 2008. All patients fitted the Dutch notification criteria; a laboratory confirmation of Q-fever and clinical presentation of fever, pneumonia or hepatitis. Patients were diagnosed by 4 different laboratories. At the beginning of the outbreak in 2007 the laboratory test most frequently used was the CFT (complement fixation test). A sero-conversion or a fourfold increase in titre, between two subsequent tests with a minimum time interval of two to four weeks, was considered positive. Later during the outbreak one laboratory used the IFA (Immuno Fluorescence Assay). This latter test distinguished between phase I en II IgM and IgG [13].

Exclusion criteria were: an unknown onset of Q-fever infection, a questionnaire completed by another person or an incomplete questionnaire. Participants younger than 18 years of age, were excluded because the questionnaire instruments were developed for adults.

### Questionnaires

All patients that agreed to participate in the Q-Quest I study, received a questionnaire that comprised two parts: the *cost and symptoms questionnaire *which collected data on demographics, self reported symptoms, co morbidity, hospitalisation, healthcare consumption, education and employment and the *Nijmegen Clinical Screening Instrument (*NCSI) [14] to measure health status.

The NCSI is based on an empirical definition of health status [15], covering physiological functioning, symptoms, functional impairment, and quality of life (Qol) as main domains. In this study we only measured the main domains symptoms, functional impairment and QoL. These main domains are subdivided into 8 sub-domains: subjective symptoms; dyspnoea emotions; fatigue; behavioural impairment; subjective impairment; general Quality of Life (General QoL); Health Related Quality of Life (HRQoL); and satisfaction with relations [14]. Consult table 1 for definitions and instruments [15-20] of the sub-domains of health status measured by the NCSI.

**Table 1:** Definitions and instruments of the health status sub-domains measured by the Nijmegen Clinical Screening Instrument

Domain	Sub-domain	Definition	Instruments
**Symptoms**	Subjective symptoms	The patient's overall burden of pulmonary symptoms	PARS-D Global Dyspnea Activity, Global Dyspnea Burden (15)
	Dyspnoea emotions	The level of frustration and anxiety a person experiences when dyspnoeic	DEQ Frustration, Anxiety (15)
	Fatigue	The level of experienced fatigue	CIS Subjective fatigue (16)

**Functional impairment**	Behavioural impairment	The extent to which a person cannot perform specific and concrete activities as a result of having the disease	SIP Home Management, Ambulation (17)
	Subjective impairment	The experienced degree of impairment in general and in social functioning	QoLRiQ General Activities (18)

**Quality of Life**	General Quality of Life	Mood and the satisfaction of a person with his/her life as a whole	BDI Primary Care (19) Satisfaction With Life Scale (20)
	Health-related Quality of Life	Satisfaction related to physiological functioning and the future	Satisfaction Physiological Functioning, Satisfaction Future (15)
	Satisfaction relations	Satisfaction with the (absent) relationships with spouse and others	Satisfaction spouse, Satisfaction social (15)

PARS-D: Physical Activity Rating Scale-Dyspnea; DEQ: Dyspnea Emotions Questionnaire; CIS: Checklist Individual Strength; SIP: Sickness Impact Profile; QoLRiQ: Quality of Life for Respiratory Illness Questionnaire; BDI: Beck Depression Inventory

The NCSI provides normative data indicating normal functioning, mild - or severe problems for each sub-domain. The NCSI contains 8 sub-domains, each expressed as a single score on its own scale. Thus eight different scales were used. The score range indicating severe problems was based on patients with COPD attending a multidisciplinary inpatient pulmonary rehabilitation program (n = 128). The key requirement for inclusion was severe problems in multiple areas of the health status. This decision was based on a three-day intake procedure, in which elaborate assessment, physiological tests and clinical interviews with seven medical disciplines were undertaken. The score range indicating normal functioning was based on a group of healthy subjects (n = 65). Scores below the 80th percentile of healthy controls indicate the score range of normal functioning. Scores above the 20th percentile of the pulmonary rehabilitation group indicate the score range of severe problems. Higher NCSI scores indicate more problems. For more details see Peters et al [14].

### Data collection

**Figure 1:**
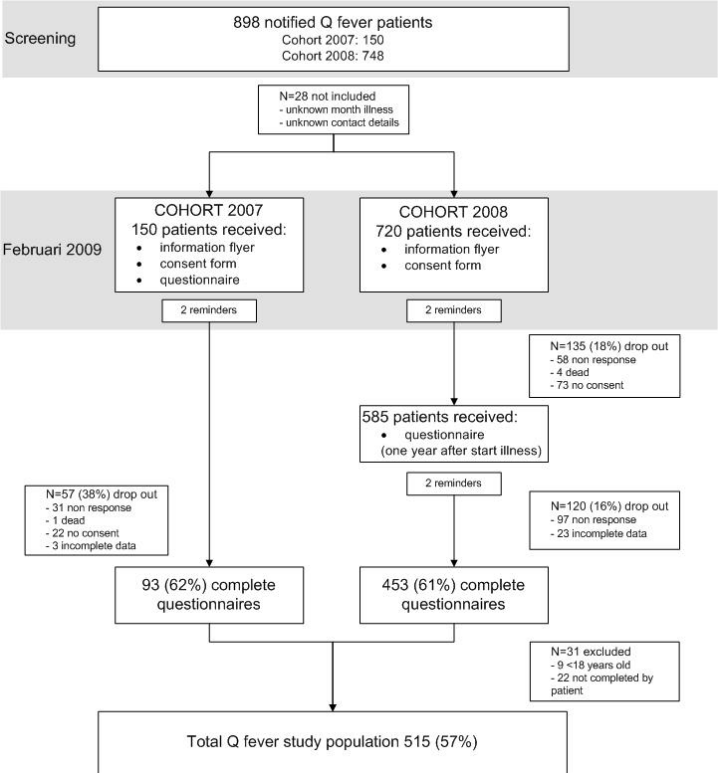
**Flowchart**. Response rate of 898 Q-fever patients with onset of disease in 2007 and 2008

In February 2009, 870 patients received a Q-Quest study information folder and a participation request form by post. Patients could state their willingness to take part in any of the Q-Quest I studies by signing the consent-form. All patients from the 2007 cohort received a Q-Quest I questionnaire (12-26 months after onset of Q-fever illness) together with the consent form in February 2009. Patients from the 2008 cohort, who had stated their willingness to participate, were mailed the questionnaire exactly one year after the month of onset of illness. If questionnaires were not returned within three weeks, patients from both cohorts received two reminders three weeks apart. See figure 1 for detailed information.

The study design and protocol were approved by the local Medical Ethics Review Committee of the Jeroen Bosch Hospital.

### Data analysis

In this study we compared the Q-fever patients NCSI scores with those of the norm groups: healthy individuals (n = 65) and the special group of severe COPD patients (n = 128).

Questionnaires were double scanned in November 2009. SPSS 15.0 for windows was used for statistical analysis. P-values were based on two tailed tests with P < 0.05 defined as significant. Chi-square test was used to compare proportions. Logistic regression and the general linear model were used to model outcomes (8 sub-domains of NCSI) for the three groups (healthy COPD-norm group and Q-fever patients), while controlling for the potential confounders: age, gender, smoking and education-level. During logistic regression we regrouped the outcomes normal, mild and severe for the 8 sub-domains into normal and abnormal (combining mild and severe). Notification data of the Municipal Health Service enabled us to compare Q-fever respondents and non responders for year of onset of illness, age, gender and hospitalisation at the acute stage of the infection. As the control groups providing the normative data for the NCSI were older than 50 years, Q-fever patients younger than 50 years of age were analyzed separately from patients older than 50 years.

For comparison of participating Q-fever patients younger or older than 50 years of age, we also looked at co-morbidity and hospitalisation. These data were unavailable for healthy individuals and COPD patients.

## Results

### Patient participation

Of the 898 patients notified in 2007-2008, 28 were excluded due to incomplete data or unknown month of onset of illness (figure 1). Of the 5 patients that died, we lacked information on the cause of death. In total 572 questionnaires were received (65.7%). Fewer men than women returned the questionnaire (responders vs. non-responders women 223/106, men 323/218 p = 0.017). The response rate was higher for patients aged over 35 (P = 0.011). After excluding participants younger than 18 years (n = 9), participants who did not complete the questionnaire themselves (n = 22) and incomplete questionnaires (n = 26), 515 questionnaires were left (see figure 1). The mean interval between the first day of illness for Q-fever patients of cohort 2007 and cohort 2008 and filling out the questionnaire was 19.6 months (SD 2.3) and 11.6 months (SD 1.0), respectively.

### Characteristics of the study population

Q-fever patients, the healthy and COPD norm group were similar with respect to gender and level of education. The characteristics of the study population are presented in table 2.

**Table 2:** Characteristics of the study population

Characteristics	Q-fever				COPD-		Healthy		Total
	Age <50		>50 yrs						
	N = 238	(%)	N = 277	(%)	N = 128	(%)	N = 65	(%)	N = 708
Gender									
Male	140	(58.8)	166	(59.9)	86	(67.2)	47	(72.3)	439
Female	98	(41.2)	111	(40.1)	42	(32.8)	18	(27.7)	269
Age									
Mean	40.4		60.3		62.5		63.5		56.7
SD	7.4		7.6		6.9		6.6		
Current smoking									
Yes	96	(40.3)	71	(26.6)	11	(8.9)	11	(16.9)	189
No	137	(57.6)	196	(73.4)	113	(91.1)	54	(83.1)	500
Education-level									
Low	56	(23.5)	97	(35.5)	62	(50.4)	20	(30.8)	235
Average	120	(50.4)	126	(46.2)	38	(30.9)	26	(40.0)	310
High	60	(25.2)	50	(18.3)	23	(18.7)	19	(29.2)	152

Q-fever patients younger and older than 50 years, Norm groups Chronic Obstructive Pulmonary Disease- and healthy individuals. Q-fever patients >50 currently smoke significantly more than COPD-controls. None of the other characteristics differ significantly (logistic regression).

### Health status

**Figure 2:**
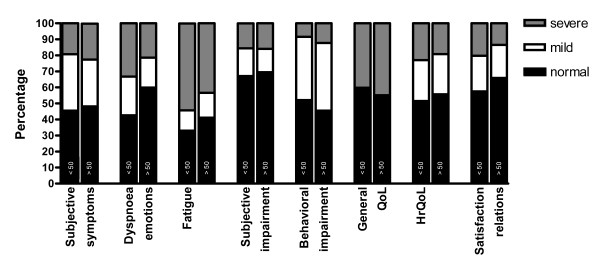
**The 8 sub-domain scores of Q-fever patients older (n = 277) and younger than 50 years of age (N = 238)**.

**Figure 3:**
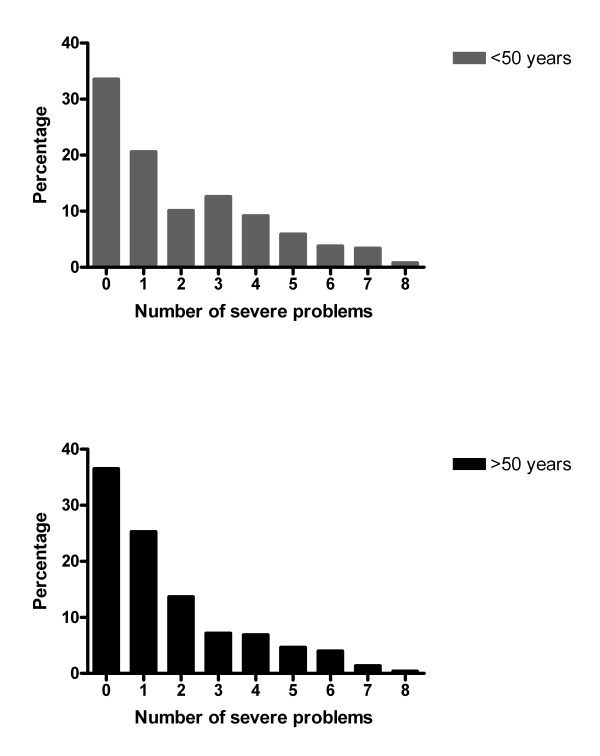
**Percentage of Q-fever patients with the number of severely affected domains of the health status**.

The long-term health status of Q-fever patients was severely affected especially for the sub-domains General QoL (44.9%) and fatigue (43.5%) (see figure 2). Almost two fifths of the Q-fever patients (38.2%) older than 50 years, had severe problems on more than one sub-domain (see figure 3). Of the Q-fever patients with abnormal fatigue, 79.5% also reported abnormal scores on subjective symptoms, 77.9% on behavioural impairment, 65.0% on HRQoL, 60.7% on dyspnoea emotions and 57.7% on General QoL.

Female Q-fever patients consistently reported abnormal functioning (mild and severe on the sub-domains of the NCSI) more frequently than males. This difference was only significant for satisfaction with relations (34.0% of the women vs. 28.1% of the men, p = 0.012).

No significant differences were found for 7 sub-domain scores between Q-fever patients older and younger than 50 years. Although the frequency with which dyspnoea was reported was similar for the age groups (45.8% >50 years n = 277 and 42.9% < 50 years n = 238) patients younger than 50 years suffered more often from dyspnoea emotions (OR 2.0, CI 1.3-3.1 p = 0.001).

In comparison to the healthy norm score, Q-fever patients showed significantly more abnormal health status (mild and severe) in 7 of the 8 sub-domains (see table 3). The worst scores were found for the sub-domains fatigue, subjective symptoms and subjective impairment. Q-fever patients had significantly lower (healthier) scores in all 8 NCSI-sub-domains, compared to the COPD-norm score.

**Table 3:** Comparison 8 NCSI sub-domains scores between Q-fever patients > 50 years and the healthy norm group

Domain and subdomain	Q-fever n = 277 (%)	Healthy control n = 65 (%)	Q-fever vs. healthy(ref)	
	n = 277 (%)	n = 65 (%)	OR (CI)	P value
**Symptoms**				
Subjective symptoms				
N	255	65		
Normal	123 (48.2)	59 (90.8)		
Abnormal	132 (51.8)	6 (9.2)	9.9 (4.0-24.5)	0.000
Dyspnoea emotions				
N	172	65		
Normal	103 (59.9)	55 (84.6)		
Abnormal	69 (40.1)	10 (15.4)	3.1 (1.4-6.8)	0.006
Fatigue				
N	207	65		
Normal	85 (41.1)	57 (87.7)		
Abnormal	122 (58.9)	8 (12.3)	9.2 (4.0-20.8)	0.000
**Functional impairment**				
Behavioural impairment				
N	277	65		
Normal	126 (45.5)	49 (75.4)		
Abnormal	151 (54.5)	16 (24.6)	3.8 (1.9-7.3)	0.000
Subjective impairment				
N	249	65		
Normal	173 (69.5)	60 (92.3)		
Abnormal	76 (30.5)	5 (7.7)	5.0 (1.9-13.4)	0.001
**Quality of life**				
General Quality of Life				
N	234	65		
Normal	129 (55.1)	51 (78.5)		
Abnormal	105 (44.9)	14 (21.5)	2.4 (1.2-4.7)	0.011
Health related Quality of Life				
N	271	65		
Normal	151 (55.7)	55 (84.6)		
Abnormal	120 (44.3)	10 (15.4)	3.7 (1.8-7.7)	0.001
Satisfaction relations				
N	252	65		
Normal	166 (65.9)	37 (56.9)		
Abnormal	86 (34.1)	28 (43.1)	0.5 (0.3-0.9)	0.040

Abnormal is a combination of mild and severe scores. Used method chi square.

The year of onset of illness, level of education and smoking behaviour had no significant influence on sub-domain mean scores. However, patients that were hospitalised (23.6% of patients older than 50 years) during the onset of illness or with underlying heart or lung disease, arthritis and depression scored significantly worse for several sub-domains (see table 4). The outcomes for patients younger than 50 years were similar.

**Table 4:** Probability of long-term impaired health-status amongst Q-fever patients older than 50 years (n = 277)

Domain	Symptoms												Functional impairment			
Sub-domain	Subjective symptoms N = 247				Dyspnoea emotions N = 166				Fatigue N = 201				Behavioural impairment N = 269			
**Factor**	**N**	**OR**	**(95% CI )**	**P value**	**N**	**OR**	**(95% CI )**	**P value**	**N**	**OR**	**(95% CI )**	**P value**	**N**	**OR**	**(95% CI)**	**P value**

Hospitalised	58	1.9	(1.1-3.6)	0.026	40	1.9	(0.9-3.8)	0.080	41	1.7	(0.8-3.4)	0.154	62	2.8	(1.5-5.1)	0.001
Diabetes	21	1.1	(0.4-2.6)	0.895	16	0.9	(0.3-2.7)	0.902	16	1.7	(0.6-4.9)	0.365	25	2.4	(0.9-5.9)	0.062
Heart disease	32	2.3	(1.1-5.2)	0.035	17	3.3	(1.1-9.3)	0.027	22	1.6	(0.6-4.2)	0.305	34	3.2	(1.4-7.3)	0.007
Lung disease	17	5.3	(1.5-18.7)	0.010	10	2.9	(0.8-10.3)	0.100	12	4.3	(0.9-19.8)	0.064	18	4.9	(1.4-17.5)	0.012
Arthritis	10	9.2	(1.2-73.9)	0.036	5	2.4	(0.4-14.9)	0.341	7	4.5	(0.5-38.4)	0.165	12	4.5	(0.9-21.0)	0.054
Depression	10	2.3	(0.6-9.2)	0.232	5	6.6	(0.7-60.6)	0.094	9	1.5	(0.4-6.1)	0.589	10	3.6	(0.7-17.1)	0.112

**Domain**	**Functional impairment**				**Quality of Life (QoL)**											
**Sub-domain**	**Subjective impairment N = 241**				**General QoL N = 234**				**Health related QoL N = 263**				**Satisfaction relations N = 245**			

**Factor**	N	OR	(95% CI)	P value	N	OR	(95% CI)	P value	N	OR	(95% CI)	P value	N	OR	(95% CI)	P value

Hospitalised	52	1.4	(0.7-2.7)	0.274	47	0.9	(0.5-1.8)	0.894	47	2.3	(1.3-4.0)	0.005	56	1.3	(0.7-2.4)	0.343
Diabetes	23	1.3	(0.5-3.2)	0.570	18	0.6	(0.2-1.6)	0.298	18	1.2	(0.5-2.8)	0.626	24	0.8	(0.3-2.0)	0.649
Heart disease	30	2.7	(1.2-5.9)	0.011	25	1.4	(0.6-3.1)	0.469	25	2.6	(1.2-5.5)	0.014	33	1.8	(0.4-1.9)	0.692
Lung disease	16	13.3	(3.7-47.9)	0.000	13	0.9	(0.3-2.7)	0.869	13	2.1	(0.8-5.7)	0.128	15	2.1	(0.7-5.8)	0.159
Arthritis	11	12.1	(2.5-57.4)	0.002	7	1.6	(0.4-7.5)	0.522	7	7.0	(1.5-32.8)	0.013	11	1.1	(0.3-4.0)	0.827
Depression	9	1.9	(0.5-7.5)	0.329	8	9.0	(1.1-74.7)	0.041	8	3.1	(0.8-12.6)	0.100	10	8.7	(1.8-42.2)	0.007

Logistic regression modelling. In all determinants "no" was the reference. Smoking and education-level were not included due to overall insignificant results.

Heart disease increased the risk for an abnormal outcome for the sub-domains subjective symptoms, behavioural and subjective impairment, HR QoL and dyspnoea emotions. Lung disease had a negative influence on the outcome of the first three aforementioned domains.

## Discussion

The present study is the largest and longest follow-up study of Dutch Q-fever patients of the 2007 and 2008 outbreaks. Using a validated questionnaire, the Nijmegen Clinical Screening Instrument (NCSI), we provided a detailed assessment of the long-term effects of Q-fever on health status 12-26 months after onset of illness. The most important finding of this study was that, in two thirds of Q-fever patients of all ages, at least one sub-domain was severely (clinically) affected up to 26 months after the initial illness. The sub-domains General QoL (44.9%) and fatigue (43.5%) were most frequently severely affected.

Published data on health status, and its sub-domains, in Q-fever patients are scarce. Hatchette reported [21] that 52% of Q-fever patients were symptomatic and had an impaired QoL 27 months after infection, with significant lower scores on five of eight domains of the Medical Outcomes Study 36-Item Short-Form Health Survey (SF-36), as compared to non-infected controls. Impaired domains were: physical pain, physical function, emotional role, physical role and social function.

In our study we found 58.9% of patients with abnormal (mild and severe) fatigue. This is similar to other publications that state 68.7% [9] five and 64.9% [8] protracted fatigue up to ten to years after infection. Unfortunately we were unable to establish if Q fever patients mainly suffered fatigue the first year and later recovered as we only had contact with patients once. The fact that we found no differences between patients of the 2007 and 2008 cohorts is suggestive of persisting complaints.

Some studies state that cytokine deregulation and immuno-modulation from persistence of C. burnetii, might be responsible [22] for prolonged fatigue, but others contradict this [23].

Other studies find prolonged impairment of the health status months after legionellosis and pneumonia. Dutch pneumonia patients had significantly affected SF-36 scores 18 months after pneumonia on the subscales physical function and general health status [24]. Survivors of a Legionnaires Disease-outbreak in the Netherlands reported 17 months after infection severely impaired SF-36-domains: physical role function, general health and vitality [25]. Up to 75.0% of patients reported fatigue [25]. Although all three infectious diseases seem to cause long-term impairment; the impaired sub-domains differ.

The severity of initial illness in general negatively influences the long-term QoL [26,27]. Similarly, the severity of the acute Q-fever symptoms predicts long-term symptoms [28]. Our study shows that hospitalised patients more often scored abnormal on the sub-domains HRQoL, behavioural impairment and subjective symptoms than those that were not hospitalised during the acute phase of illness. We consider hospitalisation to be an indicator of the severity of the initial infection. Our assumption that Q-fever patients with severe acute illness are more likely to experience long-term impaired QoL was therefore proven correct. Another study shows that patients that had been admitted to the Intensive Care Unit - regardless of the cause - have an impaired QoL (SF-36) up to 18 months [29].

General QoL (44.9%) and fatigue (43.5%) were severely affected in our study subjects. A small study on Dutch Q-fever patients that measured the one year follow-up and also used the NCSI reported a higher rate of 53% of patients with severe fatigue [12]. We suspect that the patients in that study had a higher hospitalisation rate and presented with more pneumonia than our patients. Consultation of our notification data confirmed this presumption, but the difference was marginal. Furthermore, proportionally more patients in that study might have been recruited from the local hospital's chest clinic. In the present study, we approached all patients in the region, regardless of the severity of the initial disease.

We found that heart disease increased the risk of subjective symptoms, behavioural and subjective impairment, HR QoL and dyspnoea emotions. Whereas lung disease negatively influenced the outcomes of the first three of these sub-domains.

Other authors stated that underlying heart [30,31] or lung disease [32], arthritis [33], depression [34] and diabetes [35], all had a negative effect on the health status in different sub-domains. We also found this effect, except for diabetes, but could not compare data with existing studies, as most of these studies focus on specific diseases (such as COPD) and grades of severity. We however, combined all diseases of a certain tract.

### Methodological considerations and study limitations

The NCSI is not widely used in Q-fever research. This makes comparison to other QoL-research in Q-fever difficult. The advantage of the NCSI is that it provides a detailed assessment including many domains of health status covering symptoms, functional impairment and quality of life. The NCSI provides more and specific information on sub-domains than some of the other instruments such as the SF-36. Furthermore, the availability of datasets of both a COPD and a healthy norm group for the NCSI, enabled us to compare the health status of Q-fever patients with these two groups. Such a comparison provides useful information for GPs and medical specialists in their understanding of Q-fever patients. Another advantage is that the NCSI questionnaire for the domain fatigue is based on the CIS (Checklist Individual Strength). This instrument corrects for normal fatigue [36]. As many Q-fever patients suffer from fatigue, the NCSI seemed the right choice.

The municipal health service regularly received Q-fever patient reports of continuing respiratory complaints. We therefore looked for a norm group with a known respiratory component that we could compare these Q-fever patients with. When we compared data from Q-fever patients with the NCSI norm group of COPD patients it should be realized that this is a specific subgroup of COPD patients with a severely impaired health status in multiple sub-domains. We made the choice to use this COPD norm group as we wished to compare the long-term health status of Q-fever patients (who often suffered a pneumonia initially) with another group of patients with a known impaired health status.

The healthy control group was rather small with 65 individuals all over 50 years of age. However, the number of controls provided sufficient power for us to show a large and clear difference between the groups.

Normative data of healthy subjects and those with COPD were only available for patients over 50 years of age. This was unfortunate as 46.2% of Q-fever patients were younger than 50. As we chose our method to be as strict and transparent as possible, we presented data for patients over and under 50 separately.

In at least 1.6% of the Q-fever patients in the Dutch 2007-2008 cohorts, the condition became chronic (van der Hoek et al, submitted for publication). For our study population this could potentially mean eight or nine patients with chronic Q-fever. As not all patients in our study were followed up serologically we were unable to establish if and who developed chronic Q-fever or any of its presentations such as endocarditis.

Data were collected during the early stages of the Q-fever outbreaks in the Netherlands. At that stage there was little to no media attention for these outbreaks. The general public was mostly unaware of Q-fever and the possible negative long-term outcome. Patients were not medicalised and mostly unaware. We therefore believe that our data were not negatively influenced by the media or the general knowledge of the patient of the negative long-term outcomes.

### Implications

By assessing the long-term health status of Q-fever patients of the largest outbreak in the world, we are able to describe and quantify the impact of Q-fever on patient's lives. Hospitalisation is an important predictor of severe illness, poor long-term health status outcome and long-term absence from work (unpublished data G.Morroy).

The outbreaks are continuing and Q-fever has become endemic in the area. Since symptoms could last for ten years or more [8], the burden of disease for the affected communities is likely to be considerable.

A better understanding of long-term outcomes is essential for policy makers dealing with these outbreaks. GPs and other Medical Doctors should be aware that Q-fever patients may present with long-term symptoms especially in those that were hospitalised and or with co-morbidity (heart-, lung-disease, and depression). Knowledge of these detrimental long-term outcomes should help MDs to be more supportive to these patients and refer promptly and adequately to specialist care.

## Conclusions

Our study of the largest described Q-fever cohort in the world shows a large long-term impact of Q-fever on the health status of Q-fever patients of all ages. This is but an indication of the burden of disease in the years to come considering the more than 4,000 reported Dutch Q-fever cases since 2007. Policy makers ought to take the long-term burden of disease into account, when considering measures to be taken to curb these extensive Dutch outbreaks. We recommend further research to develop adequate prevention, treatment and revalidation guidelines that might benefit these affected patients.

## Competing interests

The authors declare that they have no competing interests.

## Authors' contributions

The study idea was conceived by GM. GM and JHV participated in the design of the study. GM participated in the acquisition of the data and coordinated logistics. JHV and JBP provided previously acquired reference data. GM, MNv, JBP and HHJB carried out the statistical analysis. Data interpretation was done by GM, vMN, JBP, JHV, HHJB and WvdH. GM and MNv drafted the manuscript. All authors contributed to the critical revision of the manuscript for important intellectual content and have seen and approved the final draft.

## Pre-publication history

The pre-publication history for this paper can be accessed here:

http://www.biomedcentral.com/1471-2334/11/97/prepub
